# Persistent Cough and Speech Regression: A Multidisciplinary Case Study in a 14-Year-Old Male

**DOI:** 10.1192/j.eurpsy.2026.11176

**Published:** 2026-07-31

**Authors:** Z. Akyol, B. Çirkin, M. Yavuz

**Affiliations:** Child and Adolescent Psychiatry, İstanbul University-Cerrahpaşa, İstanbul, Türkiye

## Abstract

**Introduction:**

Chronic cough, lasting for weeks to months, is often a diagnostic challenge and can have multifactorial etiologies,including psychogenic factors.When accompanied by mutism,it raises further questions about underlying neurological or psychiatric conditions.Psychogenic cough and mutism may coexist in the context of conversion disorder or other functional neurological disorders,where psychological stress manifests as somatic symptoms.

**Objectives:**

A 14-year-old male was referred to our clinic due to progressive speech loss following persistent coughing.Symptoms began three years earlier with a barking-type cough lasting nearly six months and absent during sleep.On the fifth day of coughing, stuttering emerged and gradually progressed to severely reduced speech, limiting verbal output to a few syllables.

Pediatric and otorhinolaryngologic evaluations revealed no organic pathology.The cough appeared abruptly, interfered with speech, and evolved into stuttering and subsequent speech regression.The patient also developed school refusal, increased fears, inability to stay alone, insistence on others being present until sleep onset, and marked fear of darkness.

Aripiprazole 1 mg/day was initiated for suspected tic disorder associated with psychogenic cough and titrated to 3 mg/day, resulting in partial improvement in coughing but no speech recovery.Due to persistent symptoms, the patient was evaluated by pediatric neurology.Wake–sleep EEG showed right centro-temporal sharp-wave paroxysms during wakefulness and bilateral centro-temporal sharp-waves during sleep, while cranial diffusion MRI was unremarkable.A diagnosis of epilepsy was made, and levetiracetam 500 mg/day was started.

Psychiatric evaluation according to DSM-5 criteria resulted in diagnoses of major depressive disorder and generalized anxiety disorder, and sertraline 25 mg/day was initiated.The patient continues under multidisciplinary follow-up.

**Methods:**

Despite treatment recommendations, the patient discontinued neurology and psychiatry follow-up for 1.5 years.Seven months prior, reassessment confirmed generalized anxiety disorder; sertraline was titrated to 100 mg/day and aripiprazole adjusted to 2 mg/day.Speech therapy and neurology were reconsulted, and levetiracetam was increased to 1500 mg/day.Follow-up remains ongoing.

**Results:**

A comprehensive evaluation of the potential neurological and psychiatric etiologies underlying symptoms such as chronic cough and speech loss requires a multidisciplinary approach.In this process, to exclude potential causes such as epileptic seizures or functional neurological disorders, clinical examination should be accompanied by electrophysiological assessments, particularly EEG evaluation, which should not be overlooked

**Conclusions:**

This case underscores the importance of a multidisciplinary approach in managing complex presentations involving both neurological and psychiatric symptoms.

**Disclosure of Interest:**

None Declared

